# Molecular mechanism leading to SAHA-induced autophagy in tumor cells: evidence for a p53-dependent pathway

**DOI:** 10.1186/s12935-016-0343-0

**Published:** 2016-09-06

**Authors:** Leopold F. Fröhlich, Maria Mrakovcic, Claudia Smole, Kurt Zatloukal

**Affiliations:** 1 Molecular Pathology Laboratory, Medical University of Graz, Auenbruggerplatz 25, 8036 Graz, Austria; 2 Department of Cranio-Maxillofacial Surgery, University of Münster, Albert-Schweitzer-Campus 1, 48149 Münster, Germany; 3 Institute of Physiological Chemistry and Pathobiochemistry, University of Münster, Waldeyerstrasse 15, 48149 Münster, Germany

**Keywords:** HDACi, SAHA, Autophagy, Apoptosis, p53, HDACi, ESS-1, MES-SA, Tumor

## Abstract

**Background:**

Recent studies indicated that histone deacetylase inhibitors (HDACi), a class of anticancer agents, are in addition to their ability of apoptosis induction also capable of provoking autophagy. Promoted by the treatment of malignant uterine sarcoma cells with the HDACi suberoylanilide hydroxamic acid (SAHA), we previously demonstrated predominant dose-dependent activation of autophagy in ESS-1 cells, but prevalent induction of apoptosis in MES-SA cells.

**Methods:**

In order to extend our previous studies, SAHA-treated ESS-1 and MES-SA cells were monitored for protein expression to reveal differences in known markers of apoptosis explaining the different cytotoxic responses. Further analysis of the identified candidate protein included cell rescue experiments by gene transfer followed by subsequent screening of cells for induction of apoptosis and autophagy by immunoblotting, caspase activity as well as LC3 and MDC/PI staining. LDH release assays were performed to assess the amount of cell-mediated cytotoxicity.

**Results:**

In our search for responsible autophagic regulatory genes upstream of mammalian target of rapamycin (mTOR), we now discovered that, in contrast to MES-SA cells, a *TP53*-637C>T nonsense mutation located in the transactivating domain of the oncogenic suppressor p53 causes loss of its protein and consequently reduced PUMA induction in ESS-1 cells. Upon re-introduction of wild-type *TP53*, SAHA-treated ESS-1 cells underwent immediate apoptotic cell death as supported by upregulation of PUMA and caspase-9 as well as by activation of caspases-3 and -7 and PARP-1 cleavage. Concurrent downregulation of autophagy was noticed by upregulated mTor and phospho-mTOR expression as well as monitoring autophagosome formation employing LC3 and MDC staining. Previously, cytoplasmic master regulatory activities of the oncogenic suppressor p53 in inhibiting autophagy and triggering apoptosis were unravelled. Accordingly, p53-deficiency could explain both, the previously documented apoptosis resistance and prevailing SAHA-induced autophagy in ESS-1 cells. Using MES-SA cells with RNAi-silenced p53 expression and several p53-deficient tumor cell lines undergoing SAHA-induced autophagy, we could generally validate our finding suggesting an inhibitory role for p53 in the autophagic pathway in response to SAHA treatment.

**Conclusions:**

Conclusively, these results could identify cytoplasmic p53 protein as a molecular switch that directly mediates the cytotoxic response of SAHA and thus open new therapeutic avenues.

## Background

HDACi are a well-characterized class of cancer therapeutic agents with promising clinical activity against hematologic and solid tumors at well tolerated doses by patients [1, 2]. HDAC expression is essential in the establishment of a transcriptionally inactive chromatin structure by posttranslationally modifying histone and nonhistone acetylation sites [3]. Counteracting the effects of HDACs by inhibition of HDAC activity therefore induces chromatin relaxation which leads to altered expression of only few but important genes involved in the regulation of many disbalanced tumor cell processes as cell cycle arrest, differentiation, and apoptosis. In various tumor cell lines, treatment with HDACi most frequently induces apoptosis by sequential activation of a series of cysteine-dependent aspartate-directed proteases, called caspases [4, 5]. Nevertheless, their precise mode of function in selectively eliminating malignant cells remains unclear.

Suberoylanilide hydroxamic acid (SAHA), an inhibitor of class I and II HDACs, was previously admitted for therapeutic treatment of cutaneous T cell lymphoma [6]. As a single molecule, SAHA has broad range effects which include the inhibition of cell proliferation by blocking cell cycle progression, the suppression of angiogenesis, the induction of cellular differentiation, as well as apoptosis in tumor cells [7, 8]. Among other studies, we previously demonstrated that SAHA can, in addition to mitochondria-mediated apoptosis, also promote caspase-independent autophagic cell death [9–11] which offers an advantage in overcoming apoptotic resistant tumor cells. This coincided with decreased expression of the autophagic key molecular determinant mTOR, a master regulator of cellular metabolism which is a therapeutic target for anticancer treatment. Furthermore, phospho-S6 ribosomal protein (S6rp) which controls cell cycle progression was found to be downregulated. The studies of Gammoh et al. supported SAHA-mediated suppression of mTOR during activation of autophagy and further elaborated its downstream pathway [12]. It was found that the nutrient-sensing kinase prevents the formation of autophagy by phosphorylation and thereby inactivation of the ULK1 complex; the important role of this further central component in autophagy was previously demonstrated by ULK1-deficient cells. mTOR inactivation by SAHA thus rescues ULK1 activation and thereby induces autophagy. ULK1 (and PI3 K) complexes subsequentially activate the proper autophagy-related proteins including the ATG12-ATG5 and LC3-phosphatidylethanolamine complexes that are directly involved in formation of the mature autophagosome [13, 14]. The later involves the nucleation and elongation of the double-membrane vesicle and its fusion with the lysosome leading to the final degradation of the autophagosome content [15]. Upon conjugation to ubiquitin-like conjugation systems, cytosolic LC3 (termed LC3-I) gets incorporated to the growing autophagosome structure (then termed LC3-II) and therefore acts as a marker [16, 17].

Currently, molecular mechanisms by which SAHA modulates and thereby initiates autophagic induction upstream of mTOR remain unknown. In the absence of any observed transcriptional regulation of mTOR or upstream pathway components, the possibility remains that SAHA suppresses mTOR by interfering with the acetylation of regulatory nonhistone proteins. In this regard, the transcription of LC3 itself was found to be upregulated by SAHA, as well as downregulated by p53 which seems to support cancer cell survival under starvation conditions; nevertheless, LC3 itself is not capable of inducing autophagy [12, 18]. Previously, consistently detected upregulated expression of the class II enzyme, HDAC2, in endometrial stroma sarcoma (ESS) led us to study the therapeutic options of the HDACi SAHA [19]. Our group verified that SAHA exerts cytotoxicity on uterine sarcoma cells and significantly prevented tumor cell proliferation by increasing expression of the cell cycle kinase p21WAF1 and decreasing expression of HDAC2 and 7 in vitro [10]. The number of cells was decreased in a time- and dose-dependent manner whereby an established working dose of 3 µM SAHA significantly reduced ESS-1 cells by 80 % and MES-SA cells by 48 % after 24 h of treatment, and inhibited the G1/S transition [20]. Moreover, we documented pronounced activation of apoptosis in MES-SA uterine sarcoma cells in xenografted tumors and in vitro [21], but predominant SAHA-mediated dose-dependent autophagic cell death in malignant ESS-1 cells accompanied by decreased expression of mTOR [10]. During further experiments with combined SAHA and tumor necrosis factor-related apoptosis-inducing ligand (TRAIL) treatment, we noticed a rapidly enhanced cytotoxic effect which led to complete cell death after 24 to 48 h. In both tumor cell lines, induction of combined SAHA- and TRAIL-induced apoptosis was accompanied by upregulation of the intrinsic/mitochondrial apoptotic pathway [20]. In the present study, we wanted to further clarify the cause for the different mode of cell death in both sarcoma cell lines related to single SAHA treatment and thereby elucidate molecular mechanisms provoking SAHA-mediated autophagy.

## Methods

### Chemicals

SAHA was purchased from Alexis Biochemicals (Lausen, Switzerland). A 10 mM stock solution was prepared with dimethyl sulfoxide (DMSO) and stored at −20 °C. DMSO never exceeded a concentration of 0006 % in any experiment and therefore did not interfere with cell growth. RhTRAIL/APO-2L was purchased from eBiosciences (Vienna, Austria; 10 ng/µl) or Biomol (Hamburg, Germany; 1 µg/µl). Ampicillin, Hoechst 33258 solution, monodansylcadaverine (MDC), propidium iodide solution (PI) (1.0 mg/ml in water), and rapamycin (2.5 mg/ml solution in 2.74 mM DMSO) were received from Sigma-Aldrich (Vienna, Austria). Paraformaldehyde was acquired from Merck (Darmstadt, Germany). Inhibitors for caspase-3 (Z-DEVD-FMK), -8 (Z-IETD-FMK), -9 (Z-LEHD-FMK) and the caspase-family inhibitor (Z-VAD-FMK) were obtained from BioVision (Milpitas, CA, USA).

### Cell culture

The human uterine sarcoma cell line MES-SA [22], derived from the sarcomatous element of a mixed müllerian tumor (carcinocarcinoma), was obtained from ATCC (ATCC Nr. CRL-1976) and cultured in McCoys 5a medium (Biochrom AG; Berlin, Germany). The human ESS cell line, ESS-1 [23], was purchased from the German Collection of Microorganisms and Cell Cultures (Braunschweig, Germany) and cultured in RPMI 1640 medium (PAA; Pasching, Austria). HeLa, PANC-1, Jurkat, HL-60, and U937 tumor cell lines were obtained from the Cell Culture Core Facility of the Medical University of Graz. HeLa and PANC-1 cells were cultured in DMEM containing 4.5 g/l glucose (LifeTech; Vienna, Austria). The suspension cells Jurkat, HL-60, and U937 were grown in RPMI1640 medium (Biochrom AG; Berlin, Germany). All cell culture media were additionally supplemented with heat-inactivated fetal calf serum (10 %, v/v), 100 units/ml penicillin, 100 μg/ml streptomycin, and 2 mM of stable glutamine. Cells were cultured under standard conditions (37 °C, 5 % CO_2_, and 95 % humidity). Experiments were only conducted with cell passage numbers below 20.

### Mutation analysis of TP53 by DNA sequencing

Cells were grown to confluency and after a wash with 1× PBS, genomic DNA was extracted by incubating cells with Proteinase K digestion buffer (50 mM Tris–HCl pH 8.0, 100 mM EDTA pH 8.0, 100 mM NaCl, 1 % SDS, 0.1 mg/ml Proteinase K) at 37 °C over night. *TP53* exons were amplified from the isolated genomic DNA according to standardized primer sequences and PCR conditions of the IARC TP53 database protocol (http://p53.iarc.fr/Download/TP53_DirectSequencing_IARC.pdf). PCR products were inserted into pCR4-TOPO vector (LifeTech; Vienna, Austria) and transformed into the supplied One Shot TOP10F´ chemically competent *E. coli* cells. Transformed cells were grown on a LB plate containing 0.1 mg/ml ampicillin. Subclones were submitted for sequencing by the Sanger method for each exon (GATC Biotech AG; Cologne, Germany). The presence or absence of the *TP53* mutation was confirmed by more than tenfold re-sequencing of further ESS-1 subclones or the corresponding control region in MES-SA cells, respectively.

### Caspase activity and LDH assays

Caspase activity in the cell lysates was determined by using the Caspase-Glo 3/7 Assay (Promega; Mannheim, Germany) as previously described [24]. For individual assays, 5 × 10^3^ cells per well were seeded in 96-well plates (Corning Costar; Amsterdam, The Netherlands), incubated at 5 % CO_2_ and 37 °C, and the appropriate treatment was started 24 h later. Release of lactate dehydrogenase (LDH) into cell supernatant was measured using the CytoTox-ONE homogeneous membrane integrity assay (Promega GmbH; Mannheim, Germany) according to the manufacturer´s instructions and as previously specified [24]. For a positive control, cells were treated with a lysis solution of equal amounts of Triton X-100 and 70 % ethanol for 10 min at room temperature (RT). Results are expressed as percentage of relative LDH release compared to the lysis control. In both assays each experiment included interference controls containing no cells with the maximal concentration applied for each treatment, as well as untreated and medium controls. Caspase inhibitors were administered directly to the cells 1 h prior to the start of the treatment at a concentration of 10 µM, if required.

### Detection of autophagy/cytotoxicity by MDC/PI staining

For visualization and fluorometric quantification of autophagic cells as well as dead cells, respectively, staining with the autofluorescent drug MDC, a specific autophagolysosome marker [25], and PI was achieved as described previously [26]. 150 × 10^3^ cells were plated out on 6-well borosilicate glass plates (Asahi Glass Co.; Tokyo, Japan) and treatment was started 24 h later followed by 12 h of incubation at 5 % CO_2_ and 37 °C. Then, cells were washed once in 1× PBS and incubated for 5 min at RT with 100 µl of the cell-based PI solution added to each well and protected from light. After washing individual wells with 100 µl of 1× PBS, cells were incubated with 0.05 mM MDC in PBS at 37 °C for 60 min and protected from light. Cells were washed again in 1× PBS before they were left in 1× PBS and immediately photographed at a Zeiss confocal laser scanning microscope by using the Zeiss 1003 oil immersion lens and the LSM510 Meta software (Zeiss; Oberkochen, Germany). Images were acquired at an excitation wavelength of 514 nm for the green channel (MDC) and of 633 nm for the red channel (PI). In order to quantify MDC/PI staining, cells were monitored by fluorescence spectrophotometry (Hitachi F-2500; Tokyo, Japan) at excitation and emission wavelengths of 335 and 512 nm for MDC, respectively, and at excitation and emission wavelengths of 530 and 590 nm for PI, respectively. Incorporated MDC and PI were expressed in arbitrary units. Cells treated with rapamycin presented the positive control while untreated cells were included as a negative control. For normalization of cell numbers among different samples, MDC and PI fluorescence was adjusted to equal DNA content by Hoechst staining. After adding 1 ml of Hoechst 33258 solution (1 mg/ml) to each well, cells were incubated for 10 min and then measured at an excitation/emission wavelength of 365/460 nm. All observations were reproduced at least three times in independent experiments.

### Western blot analysis

Cell lysates and Western blots were prepared as previously described [20]. Following antibodies and concentrations/dilutions were used: rabbit anti-PUMA (1 μg/ml)/1:1000; rabbit anti-cleaved CASP-9 (1 μg/ml)/1:1000; rabbit anti Cleaved poly-ADP-ribose polymerase-1 (PARP-1)/1∶1000 (Cell Signaling; Frankfurt, Germany); purified mouse anti-human p53 (0.5 mg/ml)/1:100 (clone DO-7; BD Pharmingen; Schwechat, Austria); mouse anti-p21^WAF1^ (0.5 μg/ml)/1:2000 (Zymed; San Francisco, CA, USA); polyclonal rabbit anti-human phospho-mTOR (Ser2448) and mTOR Antibody (#2972) Antibody (Cell signaling; Frankfurt, Germany). As secondary antibodies, we used rabbit anti-mouse and swine anti-rabbit HRP-conjugated antibodies at a final concentration of 1 μg/ml/1:1000 (DAKO; Copenhagen, Denmark). Specific protein bands were visualized by enhanced chemiluminescence assay (Amersham Biosciences; Buckinghamshire, England). All Western blots were probed with a polyclonal rabbit anti β-tubulin antibody (Anti-TubA8/1:1000) (Sigma-Aldrich, Vienna, Austria) to demonstrate equal protein loading. Protein expression was quantified using Image J software.

### *TP53* gene transfer

The expression vector pCMV6-XL5 containing the human full-length cDNA for *TP53* (TP53-WT) (NCBI Acc.-No: NM_000546.2; SC119832) was obtained from OriGene (Rockville, CA, USA). Originating from TP53-WT, a control vector containing the 637C>T mutation was constructed (TP53-637C>T) by using the QuikChange II Site-Directed Mutagenesis Kit (Agilent Technologies; CA, USA). Briefly, the mutagenic primer hTP53-637C>TFwd 5′-GATGACAGAAACACTTTTTGACATAGTGTGGTGGTG-3′ was used to synthesize a mutant strand by thermal cycling using PfuUltra DNA polymerase followed by *Dpn*I restriction of the methylated parental template. Upon transformation of One Shot TOP10F′ chemically competent *E. coli* cells and selection, plasmid DNA of ampicillin-resistant colonies was isolated and submitted for sequencing in order to confirm the correct nucleotide sequence (GATC Biotech AG; Cologne, Germany). TP53-WT or TP53-637C>T were transiently transfected using Lipofectamine 2000 transfection reagent (Life Tech; Vienna, Austria) with a DNA (μg) to Lipofectamine (μl) ratio of 1:2.5. Co-transfections with the pEGFP-N1 plasmid (Clontech, GenBank acc. no. U55762; Mountain View, CA, USA) were performed to assure a high percentage of transfection efficiency (>85 %). The appropriate treatment was started 24 h after transfection.

### RNA interference

MES-SA cells were transiently transfected with 50 or 100 nM SignalSilence p53 si-RNA I or 100 nM non-targeted control si-RNA (Cell Signaling) using Lipofectamine 2000 transfection reagent. SAHA treatment was started 24 h after transfection and cells were harvested 36 h later for Western blot analysis.

### LC3 immunostaining

5 × 10^4^ cells per well were seeded in 24-well borosilicate glass plates (Asahi Glass Co., Tokyo, Japan) and incubated at 5 % CO_2_ and 37 °C. After 24 h, transfection of cells containing TP53-WT or TP53-637C>T plasmids was performed as described above and treatment of non-transfected control cells was initiated. After further incubation for 12 h, cells were washed once in 1× PBS and fixed with 3.7 % paraformaldehyde in PBS for 10 min at RT. Then, cells were washed again with 1× PBS and permeabilized with ice-cold methanol for 10 min at −20 °C followed by subsequent washing with 1× PBS. Next, cells were blocked for 30 min at RT with 1 % goat serum (DAKO; Copenhagen, Denmark) followed by incubation with a monoclonal mouse LC3 antibody (LC3-5F10, 1:100; nanoTools; Teningen, Germany) which binds to the human LC3-I (18 kDa) and LC3-II (16 kDa) forms. Subsequently, cells were washed again three times with 1× PBS before incubation with an Alexa Fluor-488 goat anti-mouse IgG (H + L) secondary antibody (Molecular Probes; Vienna, Austria) was performed for 30 min at RT under moisturized conditions. Thereafter, cells were washed again three times and were counterstained with Hoechst 33342 staining solution in 1× PBS (1 µg/ml) for 15 min at RT. After washing cells once in 1× PBS, fluorescence of LC3 positive cells was finally monitored on a Zeiss confocal laser scanning microscope using the LSM510 Meta software with an excitation/emission wavelength of 488/519 nm for green fluorescence. The mean fluorescent intensity of the measured green fluorescence was calculated and presented in arbitrary units. SAHA treated cells were included as a positive control and untreated or TP53-637C>T-transfected cells as negative control. Representative measurements, where similar results were obtained in three independent experiments, are shown.

### Statistical analysis

All values represent means of at least three independent experiments ± standard deviation (SD) of at least triplicate measurements. Statistical significance (*p*) was calculated using Student’s *t* test. A *p* ≤ 0.05 was considered statistically significant. Statistically significant differences as compared to the untreated control if not stated otherwise were indicated by asterisks (*).

## Results

**Fig. 1:**
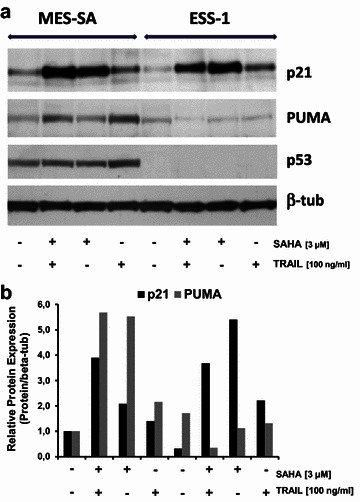
Expression analyses of key regulators of cell cycle and apoptosis in SAHA-treated uterine sarcoma cells. **a** Western blot analysis of ESS-1 and MES-SA cells treated for 8 h with 3 µM SAHA and/or 100 ng/ml TRAIL. Cell extracts were prepared, subjected to SDS-PAGE, and immunoblotted with antibodies against p21^WAF1^, p53, PUMA (23 kDa), and β-tubulin (as loading control). **b** Quantification of p21^WAF1^ and PUMA protein expression in relation to beta-tubulin expression

In a previous study, we compared the effectiveness of single SAHA versus combined SAHA and TRAIL treatment in terms of apoptosis induction [20]. 3 µM SAHA and 100 ng/ml TRAIL were established as the final most effective concentration for all further experiments. In both tumor cell lines, induction of single SAHA and synergistic SAHA- and TRAIL-induced apoptosis was accompanied by upregulation of the intrinsic apoptotic pathway verified by reduction of mitochondrial membrane potential, quantitative bivariate cytofluorometric analysis with Annexin V/propidium iodide, activation of effector caspases, and PARP cleavage. Nevertheless, in comparison, caspase-3/-7 activation was about two-fold higher in MES-SA cells than in ESS-1 cells which was consistent with the previously determined prevailing autophagy in ESS-1 cells. In order to detect any causes explaining the variable acitivation of the intrinsic/mitochondrial apoptotic pathway in ESS-1 and MES-SA cells, respectively, we analyzed the expression levels of the different cell cycle—and apoptosis-related key proteins p21^WAF1^, p53, and PUMA (p53 upregulated modulator of apoptosis) a member of the Bcl-2 protein family, by immunoblotting. As demonstrated in Fig. 1a, b, we detected essentially no irregular expression levels of the cyclin-dependent kinase inhibitor p21^WAF1^. As expected, higher p21^WAF1^ levels were observed in ESS-1 cells that received the combined treatment or single SAHA treatment whereas untreated or single TRAIL-administered cells exhibited only weakly expressed p21^WAF1^ protein in comparison. Surprisingly, however, expression of the tumor suppressor protein p53 was completely undetectable in ESS-1 cells when compared to the easily detectable and abundant expression in MES-SA cells. Furthermore, in relation to MES-SA cells lower PUMA levels were present in all treated ESS-1 samples within the range of the untreated control. This observation suggested that apoptosis resistance is caused by p53-deficiency via the p53-mediated pathway and associated with lacking PUMA upregulation in ESS-1 cells.

### Identification of a nonsense mutation in the *TP53* gene of ESS-1 cells

**Fig. 2:**
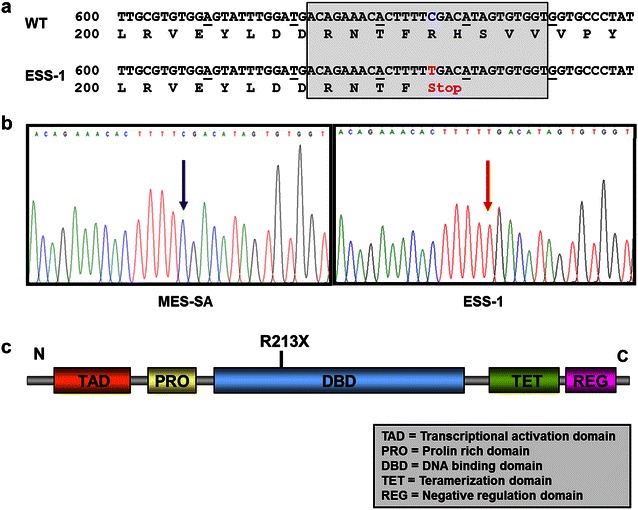
Identification of a nonsense mutation detected in the *TP53* gene of ESS-1 cells. **a** Depiction of the nucleotide sequence of exon 6 of *TP53* and the deduced encoded amino acid sequence of the p53 protein. Presented is the wildtype (WT) and the mutated sequence with a C>T transition in nucleotide position 637 of ESS-1 cells (ESS-1). The point mutation leads to the formation of a premature stop (TGA) codon at amino acid residue 213. **b** Nucleotide sequence traces of MES-SA (*left box*) and ESS-1 cells (*right box*) demonstrating the wild-type or mutated sequence (*red arrow*) presented in the *shaded box* in **a**, respectively. **c** Schematic representation of the p53 protein with its domains (not drawn to scale) and indication of the N-(N) and C-terminal **c** end. The R213X non-sense mutation is located at the N-terminal end of the DNA binding domain (DBD) of the p53 protein. As a consequence, a resulting truncated p53 protein would lack ~ 180 out of 393 amino acids at the C-terminal end

In order to verify the results of p53 immunoblotting suggesting the presence of a genomic deletion or mutation (Fig. 1), nuclear DNA was isolated from ESS-1 and MES-SA cells. However, when amplifying genomic DNA from different exonic regions of *TP53*, specific PCR fragments were present upon agarose gel electrophoresis indicating the absence of a large genomic deletion in ESS-1 cells (data not shown). As a consequence, all *TP53* exons were PCR amplified from ESS-1 and MES-SA cells, subcloned, and the nucleotide sequence was analyzed for the presence of mutations. Thereby, in ESS-1 cell derived genomic DNA, a homozygous 637C>T transition in exon 6 of *TP53* was revealed that was not present in MES-SA cells. This point mutation generates an early stop codon, resulting in a truncated p53 protein missing the C-terminal ~180 of 393 amino acids. At the protein level, the R213X non-sense mutation is located at the N-terminal region of the DNA binding domain (DBD) of p53 as depicted in Fig. 2. This finding implicated that apoptosis resistance or induction of autophagy is related to p53-deficiency due to the TP53-637C>T mutation in ESS-1 cells.

### *TP53* gene transfer restores SAHA-induced apoptosis in ESS-1 cells

**Fig. 3:**
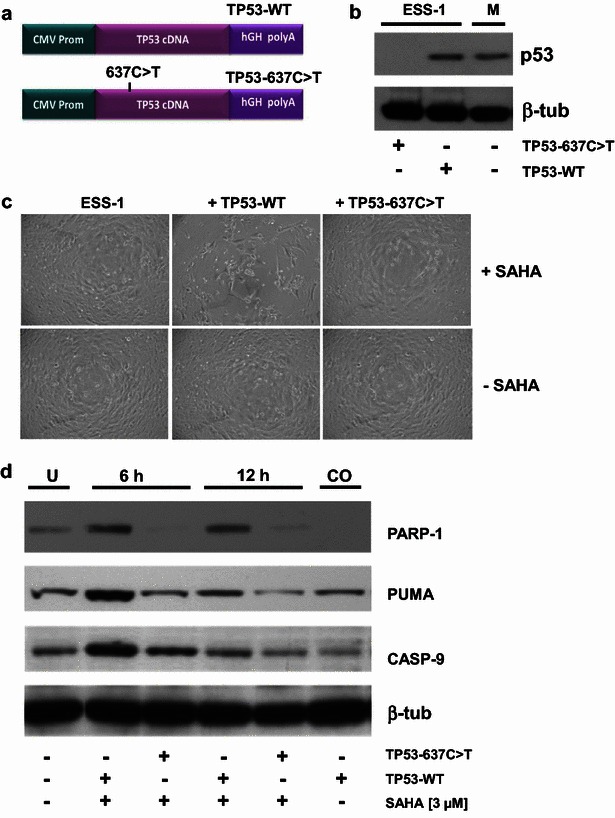
*TP53* gene transfer restores apoptosis induction by SAHA in ESS-1 cells. **a** The expression vector pCMV6-XL5 containing the CMV promoter, either wild-type (TP53-WT; *upper panel*) or the constructed 637C>T mutated (TP53-637C>T; *lower panel*) human full-length cDNA for TP53, and the signal sequence for the human growth hormone signal (hGH polyA). **b** Detection of p53 expression by Western blot in ESS-1 cells transiently transfected with the TP53-WT or -637C>T expression vectors. MES-SA cells were used as a positive control. β-tubulin was used as loading control. **c** Transient transfection of ESS-1 cells with TP53-WT and TP53-637C>T expression constructs shown in **a**. Following 3 µM SAHA treatment, TP53-WT-transfected cells underwent close to complete lysis 24 h after transfection (*upper middle panel*) while no obvious effects could be seen in SAHA-treated (*upper panel*) or –untreated (*lower panel*) ESS-1 (*left panel*) and TP53-637C>T transfected (*right panel*) control cells. (*Magnification*: 20-fold) **d** Screening for p53-induced mitochondria-mediated apoptosis by Western blot analysis. Cell extracts of ESS-1 cells that were untreated (*U*) or transiently transfected with TP53-WT, or TP53-637C>T expression vectors and were treated or non-treated (CO; 6 h) with 3 µM SAHA were electrophoresed by SDS PAGE, blotted, and monitored after 6 or 12 h with antibodies against the cleaved pro-apoptotic protein PUMA (23 kDa), caspase-9 (CASP-9; 35/37 kDa), and cleaved PARP-1 (89 kDa). β-tubulin was used as loading control

**Fig. 4:**
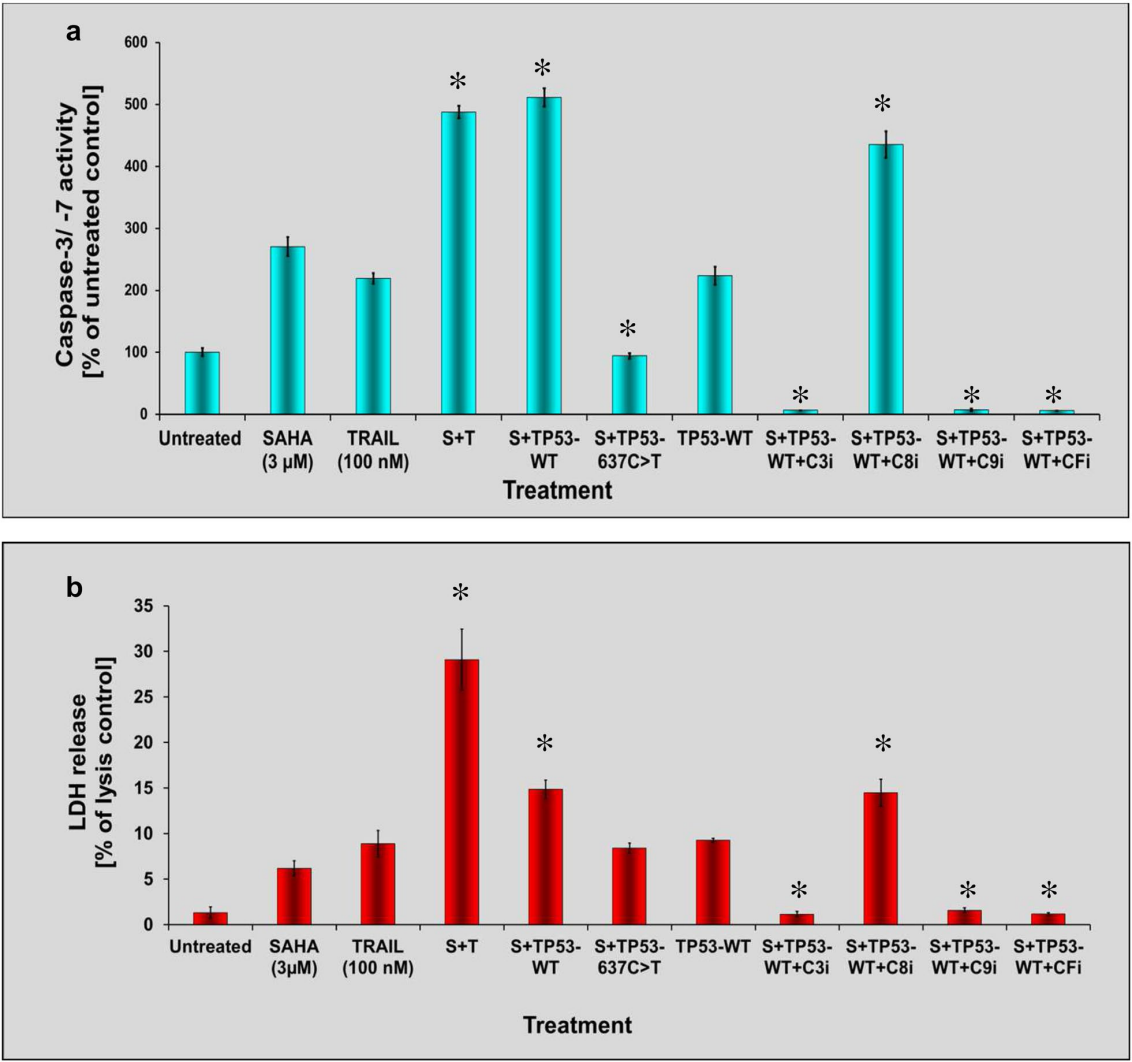
Apoptosis executioner caspase re-activation and LDH release in *TP53*-rescued ESS-1 cells. **a** Caspase-3 and -7 activation (Caspase-Glo 3/7 Assay) of TP53-WT- or TP53-637C>T-transfected ESS-1 cells that were treated with 3 µM SAHA. Different individual caspase inhibitors (C3i, C8i, C9i, CFi) were additionally applied together with SAHA to TP53-WT reconstituted cells as controls. Untreated, SAHA-, (S; 3 µM) and/or TRAIL-treated (T; 100 nM), and untreated TP53-WT-transfected cells served as further controls. **b** Measurements of LDH release by the CytoTox-ONE homogeneous membrane integrity assay of the samples analyzed in A for determination of the amount of cell cytotoxicity. Statistically significant differences compared to the SAHA-treated control were indicated in **a** and **b**

Next, we wanted to determine whether SAHA-induced apoptosis depends on the expression of a functional p53 protein (Figs. 3, 4). A full-length TP53 cDNA expression construct (TP53-WT) encoding the wildtype tumor suppressor protein and a constructed 637C>T mutated TP53 gene (TP53-637C>T) (Fig. 3a) were used to transiently transfect ESS-1 cells. This resulted in the expression of a detectable protein only in the case of the TP53-WT construct (Fig. 3b). Surprisingly, following 3 µM SAHA treatment, transfected cells underwent close to complete lysis 24 h later (Fig. 3c). This finding led to the conclusion, that p53 expression restored SAHA-induced apoptosis in ESS-1 cells. For direct confirmation of this assumption, we tested for upregulation of the pro-apoptotic proteins PUMA, caspase-9 and PARP-1 by immunoblotting (Fig. 3d). Six hours after TP53-WT-transfected ESS-1 cells were induced with SAHA, PUMA and caspase-9 were found to be expressed at higher levels in comparison to untreated or TP53-637C>T-transfected control cells. 12 h after transfection, this difference in expression declined presumably due to fast-acting reactivated apoptosis eliminating most cells by this time point. Nevertheless, for both time points sufficient cleavage of PARP-1 could be detected in cells transfected with TP53-WT, indicative for re-established apoptosis.

Subsequently, we tested whether the restoration of p53 also resulted in activation of apoptotic executioner caspases, in combination with LDH measurements, to assess the amount of apoptosis and cell-mediated cytotoxicity (Fig. 4a). Apoptosis detection of transfected cells was done by monitoring the activation of caspases-3 and -7, and comparing to those of TP53-637C>T-transferred or combined SAHA and TRAIL treated cells. The results demonstrate that upon addition of SAHA transient restoration of *TP53* full-length cDNA evoked re-induced activation of caspases-3 and -7 12 h after transfection of ESS-1 cells. The observed level of caspase induction was comparable to that obtained by combined SAHA and TRAIL treatment (~450 to 500 % of the untreated control). In contrast, single SAHA, TRAIL-treated, or solely TP53-WT transfected cells reached only about 200–300 % of untreated control levels. TP53-637C>T control-transfected cells exhibited levels comparable to the untreated control (~100 %). ESS-1 cells pretreated with caspase inhibitors (caspase-3, -9, or broad spectrum caspase family) exhibited no detectable activation of caspases-3 and -7 with the exception of caspase-8 inhibitor that is not interfering with the p53-mediated apoptotic pathway. The caspase-3/-7 peak induced by TP53-WT-transfected and SAHA-treated ESS-1 cells also coincided with a considerably increased LDH level in ESS-1 cells (~15 % of lysis control) (Fig. 4b). Nevertheless, at the investigated time-point, the levels of SAHA and TRAIL treated control cells were not yet reached (~28 % of lysis control), but were higher than that of cells supplied individually with SAHA-, TRAIL-, TP53-WT- or SAHA and TP53-637C>T (~6 to 9 % of lysis control). Conversely, caspase-inhibitor supplemented cells only reached LDH amounts which were comparable to that of untreated control cells. However, again caspase-8 inhibitor showed no significant inhibitory effect. Overall, our results confirmed p53 dependant restoration of SAHA induced apoptosis in ESS-1 cells.

### Downregulation of autophagy in *TP53*-rescued and SAHA treated ESS-1 cells

**Fig. 5:**
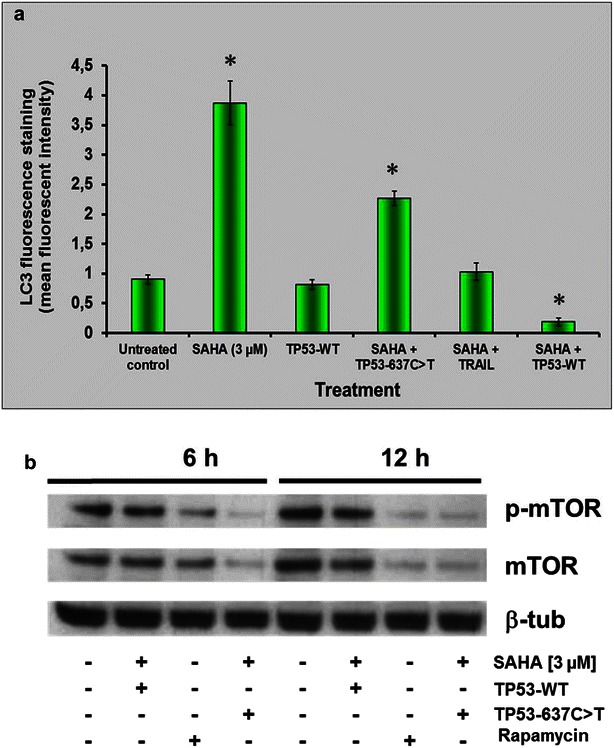
Downregulation of autophagy in *TP53*-rescued and SAHA-treated ESS-1 cells. **a** Fluorescence staining of TP53-WT resubstituted, TP53-637C>T control-transfected, or non-transfected ESS-1 cells that were treated with 3 µM SAHA with a monoclonal antibody against the autophagy-specific marker LC3. Graphs depict the mean fluorescence intensity of LC3 specific staining, 12 h after the initiation of treatment. Untreated, SAHA (3 µM)-, SAHA and TRAIL-treated (100 nM), as well as untreated TP53-WT-transfected cells were measured as controls. **b** For immunoblotting analysis with antibodies against mTOR (289 kDa) and phospho-mTOR (p-mTOR), TP53-WT- or TP53-637C>T control-transfected ESS-1 cells were analyzed 6 and 12 h after treatment with or without SAHA. Untreated or rapamycin-supplied (2 µM) cells served as controls. β-tubulin was used as loading control

**Fig. 6:**
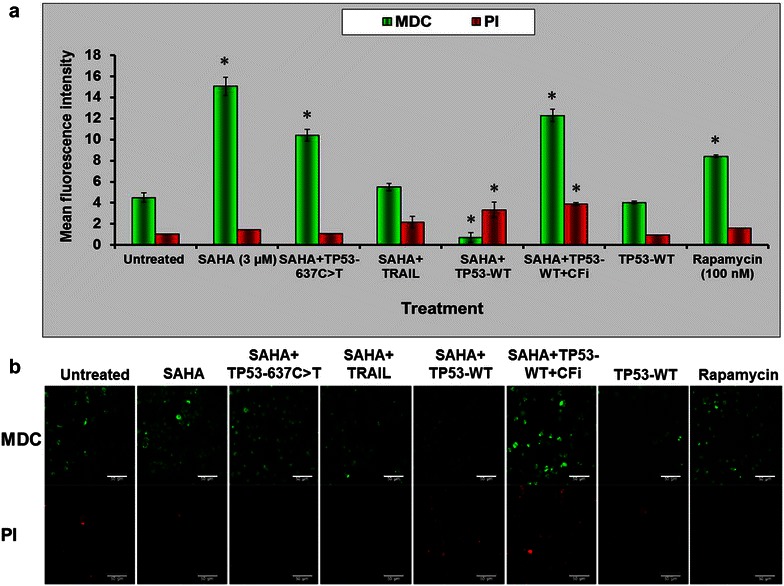
Downregulation of autophagy in *TP53*-rescued and SAHA-treated ESS-1 cells demonstrated by MDC/PI staining. **a** MDC (*green*) and PI staining (*red*) was performed of TP53-WT, TP53-637C>T, or non-transfected ESS-1 cells treated with 3 µM SAHA to quantify autophagosome formation and cytotoxicity, respectively. Untreated, SAHA, SAHA and TRAIL-treated, as well as TP53-WT-transfected cells were included as controls. Rapamycin-supplied cells were included as a reference for induction of autophagy. TP53-WT-rescued cells pretreated with 10 µM caspase family inhibitor (CFi) served as a control to monitor the influence of caspase-dependent apoptosis. **b** Representative confocal images used for quantitative autophagic and cytotoxicity measurements in **a**. At least three different images resulting from three independent experiments were used for analysing MDC (*upper panel*; *green fluorescence*) and PI (*lower panel*; *red fluorescence*) staining. The *scale bar* indicates 50 µM (*Magnification*: 40-fold)

To investigate whether restoration of p53 wildtype expression exerts an effect upon SAHA-induced autophagy in ESS-1 cells, we used different methods to monitor any induced differences in this cellular process (Figs. 5, 6). First, a specific monoclonal antibody against the autophagic marker LC3 was used for fluorescence staining (Fig. 5a). 12 h after the initiation of treatment, only basal LC3 specific staining (mean fluorescence intensity) could be detected in p53 reconstituted ESS-1 cells which received SAHA treatment in comparison to only SAHA-treated cells (~0.2 vs. ~3.9 arbitrary units) or SAHA and TP53-637C>T treated control cells (~0.2 vs. ~2.3 arbitrary units). Nevertheless, even in the absence of SAHA-induced autophagy, untreated, as well as TP53-WT treated control cells exhibited about fivefold higher levels of LC3 staining compared to *TP53*-transfected cells (~0.2 vs. ~1 arbitrary units). Also in SAHA- and TRAIL-induced control cells, in which apoptotic cell death is prevailing [20], LC3 staining still reached a level of ~1 arbitrary units. For mTOR immunoblotting analysis, TP53-WT transfected cells were investigated after supplying them with 3 µM SAHA for 6 and 12 h (Fig. 5b). Consistent with basal induction of autophagy for both timepoints, bands comparable to the untreated control could be detected for the serine/threonin protein kinase mTOR and phosphorylated mTOR (p-mTOR); in contrast, rapamycin-treated or TP53-637CT-transfected control cells that were supplemented with SAHA showed decreased mTOR or p-mTOR signals indicative for induction of autophagy.

Next, we additionally employed MDC and PI staining to quantify and depict *TP53* transfected ESS-1 cells regarding autophagosome formation and cytotoxicity (Fig. 6a, b). Upon SAHA treatment, TP53-WT-rescued cells displayed only marginal mean fluorescence intensity staining for MDC in comparison to untreated or TP53-637C>T-transfected control cells (~0.5 vs 4 arbitrary units); single SAHA or rapamycin-induced positive ESS-1 control cells, however, reached ~16 or 30-fold higher levels of MDC staining, respectively. Surprisingly, pretreatment of TP53-WT-transfected ESS-1 cells with the broad spectrum caspase family inhibitor suppressing caspase-dependent apoptosis also led to considerable upregulation of autophagosome formation (~11 arbitrary units). TP53-WT transfection with or without caspase-inhibitor addition provoked the highest observed cytotoxicity as indicated by PI staining which was ~fourfold higher than in all other control cells upon 12 h of treatment. Representative confocal images of the cells used for MDC as well as PI fluorescence measurements in Fig. 6a are presented in Fig. 6b. Taken together, we showed that p53-restored cells displayed diminished autophagic induction.

### RNAi silencing of p53 expression induces autophagy in SAHA-treated MES-SA cells

**Fig. 7:**
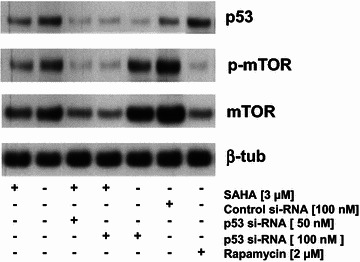
Si-RNA mediated knockdown of p53 expression in MES-SA cells. Immunoblotting analysis was used to monitor the amount of silencing of p53 expression, and the resulting mTOR (289 kDa) and p-mTOR expression indicating autophagic induction 36 h after transiently transfecting MES-SA cells with si-RNA for p53 (50 and 100 nM) or non-targeted control si-RNA (100 nM). SAHA-treatment (3 µM) was started 12 h before analysis. Untreated, only SAHA-treated, or rapamycin-supplied cells served as additional controls. β-tubulin was used as loading control

In contrast to ESS-1 cells lacking p53 expression, we wanted to test whether downregulation of p53 expression in p53-proficient cells will induce autophagy. Therefore, we employed RNA interference in MES-SA cells to silence its p53 expression (Fig. 7). Besides p53, mTOR and p-mTOR expression were examined by immunoblotting analysis. Transient transfection of MES-SA cells with p53 si-RNA demonstrated partially reduced p53 expression which was not present in untreated or control si-RNA transfected cells. Diminished p53 expression furthermore correlated with reduced expression of both investigated mTOR proteins in SAHA-treated MES-SA cells, comparable to rapamycin-treated control cells and thus indicated enhanced induction of autophagy. Untreated, SAHA-supplemented, or control si-RNA transfected cells lacked or showed only insignificantly decreased mTOR and p-mTOR signals, however. Therefore, this experiment suggested that diminished p53 expression could also lead to increased autophagic induction in MES-SA cells undergoing SAHA treatment.

### Monitoring of *TP53*-rescued tumor cell lines for apoptosis and autophagy induction

**Fig. 8:**
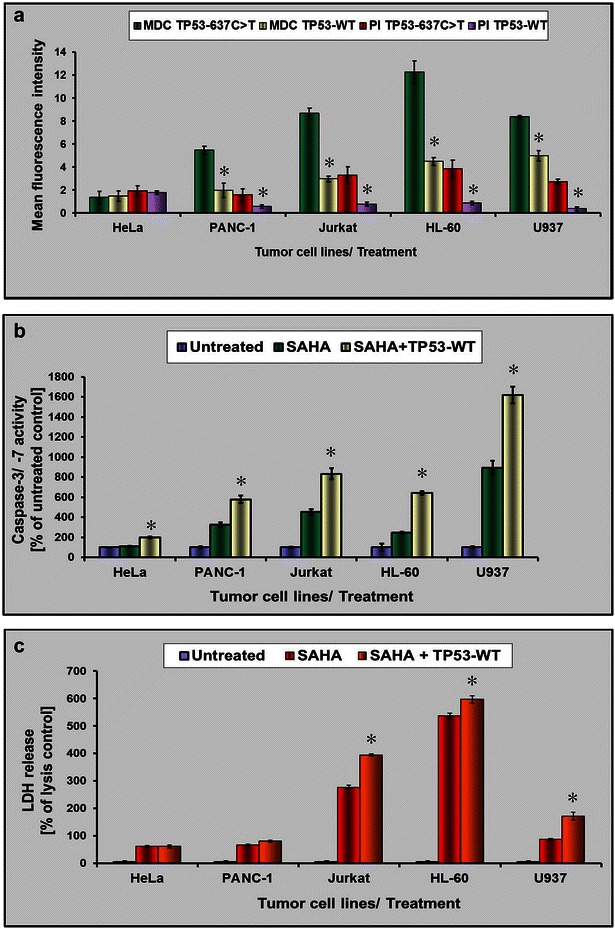
Monitoring of *TP53*-rescued tumor cell lines for apoptosis and autophagy induction. **a** A TP53-proficient (HeLa) and several *TP53*-mutant and SAHA-responsive tumor cell lines (PANC-1, Jurkat, HL-60, and U937 cells) were transfected either with TP53-WT or with TP53-637C>T, respectively. After 24 h of 3 µM SAHA treatment, MDC and PI fluorescence was quantified in order to monitor resulting changes in autophagy and cytotoxicity. **b** To assess apoptotic activity in TP53-WT reconstituted tumor cell lines as described in **a**, caspase-3 and -7 activation (Caspase-Glo 3/7 Assay) was measured upon 3 µM SAHA treatment for 24 h and compared to untreated, or only SAHA-treated cells. **c** Additionally, the amount of LDH release was determined from cells used for the experiment in **b**. Statistically significant differences compared to the SAHA-treated control are indicated in **a**–**c**

Next, we wanted to generally validate a role for the tumor suppressor protein p53 in switching between SAHA-mediated apoptosis and autophagy due to its functional status or cytoplasmic presence in the cell. Therefore, upon *TP53* reconstitution and administration of 3 µM SAHA, several *TP53* mutant and SAHA-responsive cell lines, which have been previously reported to undergo autophagy, were screened for their mode of cell death [27–30]. HeLa cells, that undergo SAHA induced apoptosis and possess a wildtype p53 protein have been included as a control [31]. As demonstrated in Fig. 8a, with the exception of HeLa control cells, the level of autophagy was downregulated in all investigated p53-deficient tumor cell lines (PANC-1, Jurkat, HL-60, U937) after 24 h indicated by less detectable MDC staining; mean fluorescence levels were found to range from ~31 to ~65 % lower in comparison to non-transfected but also SAHA-treated cells. In line with this finding, also the amount of dead cells was found to be significantly reduced for ~53 to 85 % for all *TP53*-*WT* transfected cell lines, when compared to control cells, as indicated by PI staining. Conversely, when apoptosis was monitored at this time point, the tumor cell panel exhibited ~76 to 157 % higher levels of caspase-3/-7 activation (Fig. 8b). Furthermore, equal (HeLa cells) or slightly higher LDH levels ranging from 11 to 97 % were detected when TP53-WT was additionally transfected in comparison to only SAHA-treated tumor cells after 24 h (Fig. 8c). Conclusively, these analyses let us presume a general important role for p53 as a mediator in SAHA-driven apoptosis and autophagy.

## Discussion

In this study, we searched for the cause of varying cellular responses elicited by two human uterine sarcoma cell lines upon treating them with the HDACi SAHA. By screening several important pro-apoptotic key molecules, we found that expression of the tumor suppressor protein p53 was completely undetectable in ESS-1 cells when compared to the easily detectable and abundant expression in MES-SA cells. Furthermore, only basal PUMA levels were present in relation to MES-SA cells. Irrespective of absent p53 expression, the promising anti-cancer compound SAHA increased expression of the cell cycle kinase inhibitor p21^WAF1^ which downregulates tumor cell proliferation via cell cycle arrest in the G1/S phase. This observation confirms a previous study which reported, in contrast to normally tightly p53-controlled expression, the possibility of p53-independent activation of the p21^WAF1^ promoter by direct SAHA-mediated activation of SP1 sites [32]. Overall, this finding was in consistency with prevailing induction of autophagic cell death in ESS-1 cells [10] and the predominant apoptotic response in MES-SA cells provoked by SAHA as previously elaborated [20, 21]. Therefore, we searched for the molecular mechanism in which the absence of p53 expression originated. We detected a homozygous 637C>T transition in exon 6 of the *TP53* gene in the ESS-1 cell line which results in the formation of a premature stop codon. Since codon 213 consists of a CpG dinucleotide, which is a target for cytosine methylation, the nonsense mutation at codon 213 could have occurred in vitro in this cell line as the result of endogenous deamination of 5-methylcytosine to thymine. Upon database screening, we were also able to confirm this mutation by an entry in the COSMIC (catalogue of somatic mutations in cancer) database for human immortal cancer cell lines (http://www.cancer.sanger.ac.uk/cancergenome/projects/cell_lines/). Moreover, this mutation has been verified and annotated for endometrial carcinoma [33]. The R213X non-sense mutation, which is not present in MES-SA cells, is located at the N-terminus of the DNA binding domain (DBD) of p53 and leads to a truncated protein that is missing the last ~180 of 393 amino acids. Although we used a monoclonal human-specific p53 antibody (clone DO-7) that recognizes an epitope between amino acids 1–45 of wildtype as well as mutant forms of p53, we were not able to detect the presumptive truncated p53 protein in our experiments. In support of our finding, also ovarian cancer patients possessing the R213X mutation exhibited null type mutations that could not be detected by immunohistochemical staining for unknown reasons [34].

The apoptosis-inducing functions of p53 are exerted in two ways: one is through the transcriptional activation of pro-apoptotic proteins such as Puma, Noxa and Bax, the other is via direct physical and functional interaction with members of the Bcl-2 protein family that cause mitochondrial membrane permeabilization. The identified homozygous p53 mutation thus clearly explains apoptosis resistance in ESS-1 cells as in the case of complete p53-deficiency, no physical interactions or transcriptional activities can take place at all. Alternatively, in case of undetectable p53 protein, it was previously reported that the R213X mutation disrupted the efficiency of p53 transactivation by its inability to bind consensus binding sequences of p53 in the regulatory region of the p21WAF gene [35]. In addition, this mutation was also found to be a dominant negative p53 variant which suppresses the endogenous wild-type function [36]. Beyond that, we hypothesized that p53 could be the missing link leading to either SAHA-induced autophagy in the absence of the p53 protein or preferential SAHA-stimulated apoptosis when a functional wild type molecule is present in the cell. In support of this, in addition to its many other tumor suppressing activities, p53 has been identified as a major regulator of autophagy in recent time [37–40]. Autophagy induced by p53 may facilitate p53's cell cycle arrest activities, such that autophagy mediates the selective degradation of damaged molecules and organelles in order to provide an energy source for the damage repair process and promote ‘cell healing’. Alternatively, when the extent of damage is beyond repair, autophagy may act to synergize with accelerated cell death in response to p53 activation. While the autophagy-promoting activity of p53 requires the presence of p53 in the nucleus, associated with transcriptional activity, the autophagy-suppressing function of p53 was found to completely depend on its cytoplasmic presence without transcriptional dependence. Both, autophagy-promoting as well as -inhibiting activities of p53, engage the mTOR signaling pathway which in response to genotoxic or metabolic stress cross-talks with p53 in a coordinated fashion [41]; thus, both signaling machineries regulate cell growth, proliferation, and death together. This mechanism of p53-induced autophagy involves activation of 5′ AMP-activated protein kinase (AMPK) as well as the tuberus sclerosis complex kinases, TSC1 and TSC2, which finally inhibit mTOR kinase. Indeed, after rescue of ESS-1 cells with wildtype *TP53* we found in our experiments that the balance between predominant autophagy and apoptosis was directed towards prevailing apoptosis and basic autophagy; this observation was supported by upregulation of PUMA, as well as by activation of the apoptosis initiator and executioner caspases 9,-3 and -7, and finally by PARP-1 cleavage. In contrast, downregulation of autophagy was supported by mTOR/phosphor-mTor immunoblotting, staining with the autophagic marker LC3 as well as specific autophagosome staining with MDC [25]. These results could identify p53 as a molecular switch that directly mediates the response of SAHA by either executing pro-apoptotic signalling; mechanistically this could possibly be accomplished by direct acetylation of the protein or by promoting the formation of autophagy upon the absence of p53 in the tumor cell. Accordingly, a study demonstrated that complexes constituted by acetylated p53 as well as acetylated histones and coactivators were held responsible for HDACi-induced apoptosis in HepG2 cells [42]. If proven true, these findings furthermore might have immediate implications in the choice of cancer therapeutics and make our experimental system interesting for further molecular analysis regarding SAHA-provoked cell death regulation.

## Conclusions

Taken together, we conclude that in ESS-1 and MES-SA cells and at least in a subset of tumor cell lines in general, p53 could be essential in mediating SAHA–induced apoptosis, while absence or degradation of p53 in the cytoplasmic compartment could lead to activation of a SAHA-mediated autophagic pathway. Overall, our data provide encouraging evidence for an inhibitory role of a p53-driven autophagic response elicited by the SAHA pathway in tumor cells and the requirement of p53 for undergoing SAHA-induced apoptotic cell death. Thus, mutant p53 re-activation using a well-tolerated small molecule inhibitor or inhibitors of MDM2-mediated proteasomal degradation might help to restore p53-dependent apoptosis and tumor suppression. As nonsense TP53 mutations of which the 637C>T mutant is found most often are even more common in human tumors than TP53 missense mutations (listed in the UMD TP53 muatation database; http://p53.free.fr), their restoration by drugs will be of significance but require different approaches. Currently, aminoglycosides (e.g. gentamicin and G418), can achieve read-through of the 637C>T transition leading to expression of full-length p53, however due to their toxicity further screening for less toxic compounds is necessary [43]. The resulting molecular shift from autophagy towards apoptosis could then help in the efficient removal of tumor cells in affected patients [44]. In addition, in a subset of tumor cells where inhibition of autophagy causes non-apoptotic cell death, the therapeutic potential of autophagy targeting in combination with SAHA therapy could be employed [45].

## Abbreviations

DMSO: dimethylsulfoxide
ESS: endometrial stroma sarcoma
HDAC(i): histone deacetylase (inhibitor)
LDH: lactate dehydrogenase
MDC: monodansylcadaverine hydroxamic acid
mTOR: mammalian target of rapamycin
p53: tumor suppressor protein 53
PBS: phosphate buffered saline
PI: propidium iodide
PUMA: p53 upregulated modulator of apoptosis
RNAi: RNA interference
RT: room temperature
SAHA: suberoylanilide hydroxamic acid
TP53: gene encoding p53
TRAIL: tumor necrosis factor-related apoptosis-inducing ligand
